# Surgical Excision of a Symptomatic Thoracic Nerve Root Perineural Cyst Resulting in Complete Resolution of Symptoms: A Case Report

**DOI:** 10.7759/cureus.1343

**Published:** 2017-06-12

**Authors:** Zaid Aljuboori, Alae Yaseen, Jessica Simpson, Maxwell Boakye

**Affiliations:** 1 Neurosurgery, University of Louisville School of Medicine; 2 Pathology, University of Louisville School of Medicine; 3 University of Pikeville Kentucky College of Osteopathic Medicine

**Keywords:** cyst, meningeal, nerve, perineural, root, spinal, tarlov

## Abstract

Tarlov (perineural) cysts of the nerve root are common and usually incidental findings during magnetic resonance imaging (MRI) of the lumbosacral spine. There are a few case reports where symptomatic thoracic perineural cysts have been described in the literature. We report a case of a high thoracic nerve root perineural cyst that failed conservative therapy, requiring surgical intervention. Our patient presented with radicular symptoms involving the left hand. Imaging workup revealed a cystic lesion of the left T1 nerve root at the level of the foramen. Surgical resection resulted in significant improvement in patient symptoms, and pathology revealed a perineural cyst. We conclude that a thoracic perineural (Tarlov) cyst can be symptomatic by causing nerve root compression and can be mistaken as a nerve root sheath tumor on imaging. Surgical treatment can be curative.

## Introduction

Spinal extradural perineural cysts were initially described by Tarlov [1]. They are classified as type II meningeal cysts [2], most frequently found in the sacrum and very rarely in the thoracic spine. They are also known as spinal nerve root diverticula or ‘Tarlov perineural cysts’ [3]. Incidental cysts are usually managed expectantly, but the treatment of symptomatic cysts is controversial [4]. Here, we present a case of a symptomatic thoracic nerve root perineural cyst treated with surgical excision.

## Case presentation

**Figure 1:**
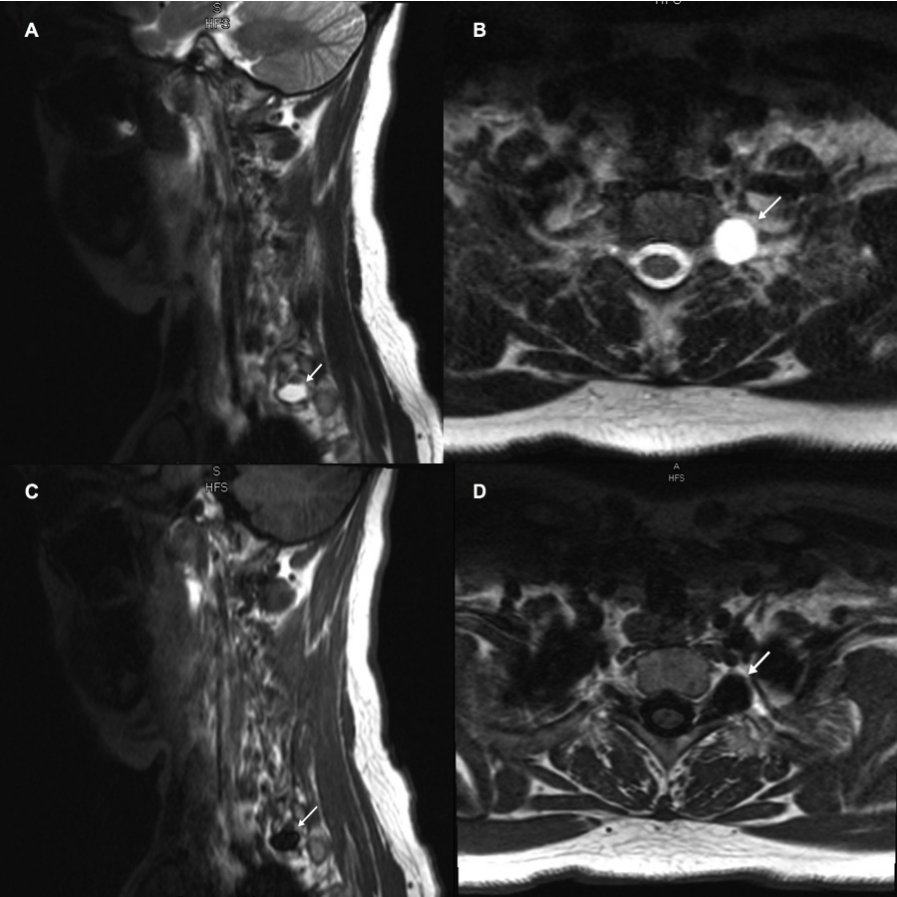
MRI of cervical spine without contrast [A] T2 weighted sagittal images show a hyperintense lesion (white arrow) that originates from the T1 nerve root and extends to the T1-T2 left neuroforamen; [B] T2 weighted axial images show a hyperintense lesion (white arrow) that originates from the T1 nerve root and extends to the T1-T2 left neuroforamen; [C] T1 weighted sagittal images show a hypointense lesion (white arrow) that originates from the T1 nerve root and extends to the T1-T2 left neuroforamen; [D] T1 weighted axial images show a hypointense lesion (white arrow) that originates from the T1 nerve root and extends to the T1-T2 left neuroforamen.

Our patient, a 39-year-old, white female with past medical history of anxiety and chronic obstructive airway disease (COPD) presented with five weeks’ history of left upper extremity pain and a burning sensation. It was progressive in nature and involved the medial arm, shoulder, and lateral thorax. The patient denied any trauma. The intensity of the pain resulted in multiple admissions to the emergency room (ER) with discharge on narcotics for pain control. Later, during the course of her disease, she developed weakness in the left hand. The patient's neurological exam was unremarkable except for decreased sensation to light touch in the distribution of left C7/C8 dermatomes, mild weakness of left triceps, and significant weakness of left-hand grip. The patient had a cervical spine MRI, which showed a 1.25 x 1.25 cm homogenous cystic mass involving the left T1 spinal nerve within the neuroforamen. It appeared hypointense and hyperintense on T1W and T2W imaging respectively (Figure 1).

**Figure 2:**
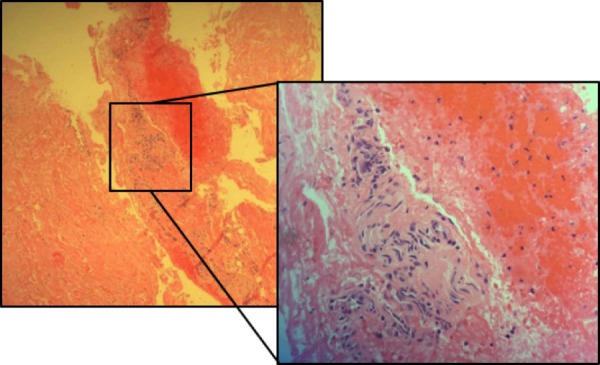
Histopathology sections The section shows fragments of benign fibrous tissue with nonspecific changes [hematoxylin and eosin (H&E) X 4]. The enlarged section shows an area of tissue of nerve fibers bordered by blood and fibrin deposition with scattered neutrophils. No active inflammatory process was noticed. No atypical cells were seen either (H&E X 20).

**Figure 3:**
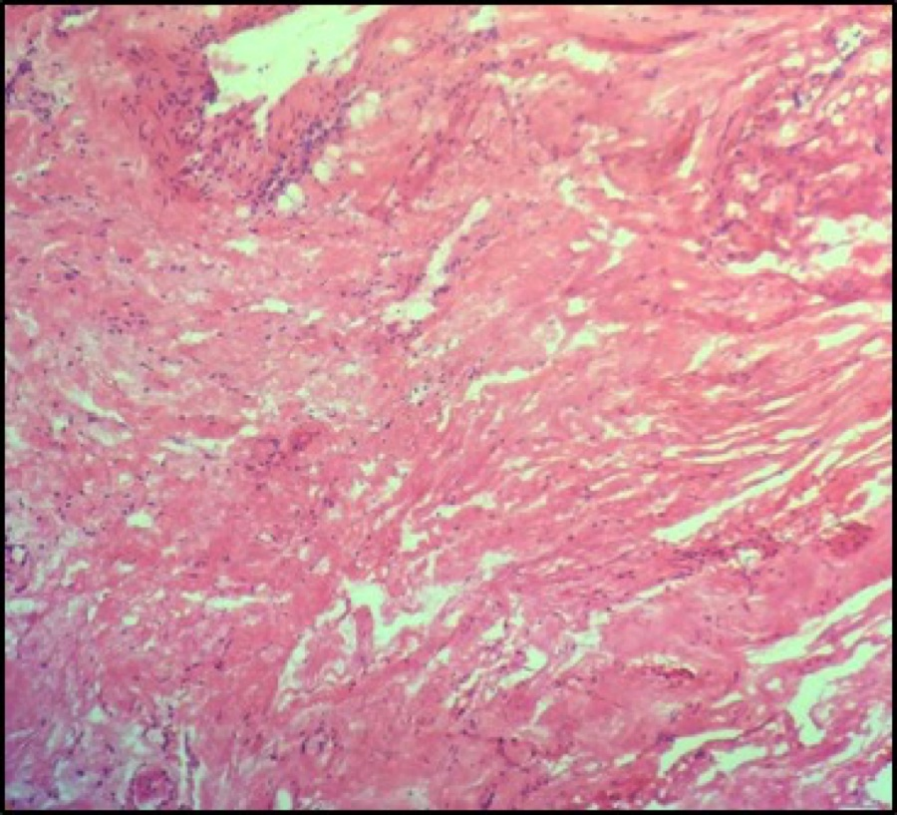
Histopathology section The section shows an area of fibrous tissue with fibrin deposition and minimal scattered inflammatory cells infiltrate (H&E X 20).

Initial conservative therapy failed to control the patient’s symptoms, and given the new weakness of the left hand, which affected her daily activities, we decided to intervene surgically. The patient underwent T1/T2 left-sided partial laminectomy, and a total facetectomy with excision of the cyst followed by posterolateral fixation of C7-T3. Intraoperatively, the lesion appeared gray in color with a white capsule. Before opening the capsule, we used intraoperative electrical stimulation to identify and preserve the nerve root. Stimulation of the cyst did not produce any positive responses. At this point, we opened the capsule and a clear cerebrospinal fluid (CSF)-like liquid came out from the cyst. After resection of the cyst, the underlying nerve root was readily identifiable using electrical stimulation. The final pathology results showed fibrous tissue with nerve tissue, which is typical for perineural cysts (Figures 2-3). Postoperatively, the patient experienced significant improvement in her symptoms, represented by resolution of the shooting pain and burning sensation, as well as improved left-hand strength.

## Discussion

Perineurial cysts, also known as Tarlov cysts, were first described in 1938 by the neurosurgeon Isadore M. Tarlov during an autopsy of the lower spine [1]. He described them as nerve-root membranes that contain fluid collections with restricted communication with the CSF in the thecal sac. These cysts form only on dorsal roots because only the cell bodies of sensory neurons migrate out from the spinal cord during embryonic development, leaving behind a sleeve of dura and subarachnoid space [1, 5-6].

The origin of these lesions is unclear, with some evidence supporting inflammation within the subarachnoid space; traumatic hemorrhage that leads to blockage of the venous drainage of the perineurium and epineurium, leading to hemosiderin deposition that can result in the development of these cysts; congenital diverticula from persistent embryonic fissures; or hydrostatic CSF pressures [5,7]. It has also been suggested that the restricted communication with the CSF in the thecal sac results in a ball-valve mechanism that permits one-way entry of CSF into the cyst during systolic pulsations, with no exit during diastole [7].

The lumbosacral spine is the most common location for these cysts, with a prevalence range from 1.5% to 2.1% on lumbosacral MRI images [5]. Symptomatic cases are rare, constituting less than 1% [7]. It is predominant in females, by up to 84% as reported in one study [5]. Histologically, Tarlov cysts are characterized by the presence of nerve fibers within the fibrous tissue of the cyst wall [5,7]. This is important since an intraoperative frozen section may reveal nerve elements; however, this is differentiated from a normal nerve because of the absence of positive response to electrical stimulation.

A majority of these cysts are asymptomatic and clinically insignificant [4], though large cysts can compress and damage nerve roots, the spinal cord, or surrounding bone. Radicular neuropathic pain caused by the compression of the nerve root that bears the cyst is the most common symptom [4,7]. Because of their rarity, cervicothoracic Tarlov cysts may masquerade as tumors (e.g., nerve root sheath tumor) [7].

Published treatment options for apparently symptomatic Tarlov cysts include perineural cyst aspiration with or without injection of the cyst with blood or fibrin sealant [7]. Although the perineural injection of a glucocorticoid or local anesthetic agent can be used as a treatment method sometimes, evidence indicates it provides nothing but temporary relief. However, such procedures can be used to confirm that the cysts are the cause of the symptoms.

When conservative measures fail and quality of life is significantly impacted, authors have advocated neurosurgical treatment, which usually involves a small laminectomy with cyst wall fenestration with or without packing the cyst cavity with autologous fat or excision while avoiding neural tissues [7-8]. Large case series showed complete or substantial improvement in more than 80% of patients who underwent surgery [9-10].

## Conclusions

Thoracic perineural (Tarlov) cysts are rare but they may present in a similar fashion to nerve root sheath tumors. Surgical treatment can be curative, but recurrence is possible.
